# A proteomic analysis of differentiating dopamine neurons derived from human embryonic stem cells

**DOI:** 10.1080/19768354.2019.1595140

**Published:** 2019-04-11

**Authors:** Joohyun Ryu, Byoung Chul Park, Do Hee Lee

**Affiliations:** a Department of Cellular and Molecular Biology, The Hormel Institute, University of Minnesota, Austin, MN, USA; b Disease Target Structure Research Center, Korea Research Institute of Bioscience and Biotechnology, Daejeon, Korea; c Department of Bio and Environmental Technology, Seoul Women’s University, Seoul, Korea

**Keywords:** Human embryonic stem cells, dopamine neuron, proteome, cytoskeletal proteins

## Abstract

Human embryonic stem cells (hESC) are being exploited for potential use in cell transplantation due to their capacity for self-renewal and pluripotency. Dopamine (DA) neurons derived from hESC represent a promising source of cell replacement therapy for Parkinson’s disease (PD). While gene expression on the transcriptome level has been extensively studied, limited information is available for the proteome-level changes associated with DA neuron differentiation. Here we analyzed the proteome of differentiating DA neurons to search for the potential biomarkers to assess the efficiency of differentiation. Although the proteome profile of DA neurons did not exhibit significant changes, a number of cytoskeletal proteins including nuclear lamin, tropomyosin 1, and myosin light chain 1 were specifically up-regulated during differentiation. Expression analysis of the respective genes was also consistent with the proteome results. In addition, these differentially expressed proteins form protein interaction network with several PD-related proteins suggesting that they may play roles in PD pathogenesis as well as the maturation of DA neurons.

## Introduction

Human pluripotent stem cells are regarded as one of the most promising sources of cell replacement therapies for many incurable diseases. PD is a model disease where a stem cell-based approach is considered as a viable treatment option (Sonntag et al. 2018). Recent technical progress led to the generation of functional DA neurons from hESC and human induced pluripotent stem cells (hiPSC) for animal studies and future clinical trials (Rhee et al. 2011). Induced pluripotent stem cells from the somatic cells of patients offer a possibility for autologous cell therapy that could circumvent immunogenicity and ethical issues. However, issues including possible tumorigenesis, low reproducibility and difficulties in cell amplification still need to be addressed before hiPSC can be successfully applied for clinical purposes. In addition, hiPSC derived from patients with degenerative diseases likely retain pathological features. In fact, the gene expression profile of induced DA neurons derived from PD patients differs from that of the control primary midbrain DA neurons (Xia et al. 2016).

The advantages of non-autologous cells as a source for cell therapy include the uniform quality, high productivity and availability of various cell types (Yasuhara et al. 2017). A recent study showed that DA neurons derived from hESC possess functional properties and efficacy comparable to those of human fetal neurons (Grealish et al. 2014). Continuous efforts are being made to improve the differentiation efficiency of hESC-derived DA neurons and to suppress alternative lineages (Lim et al. 2015). Moreover, attempts to minimize the immunogenicity that hampers availability of hESC have also been made. In addition, somatic cell nuclear transplantation-derived hESC is considered as an alternative option to generate autologous cells (Tachibana et al. 2013). Efficient generation of functional DA neurons from hESC usually requires morphogens e.g. sonic hedgehog (Shh) and a cocktail of growth factors including basic fibroblast growth factor (bFGF), brain-derived neurotrophic factor (BDNF), and glial cell-derived neurotrophic factor (GDNF) in addition to feeder cells (Sonntag et al. 2018).

*In vitro* differentiated DA neurons have been extensively analyzed for gene expression on the transcriptome level as well as functionality to verify the characteristics of neuronal cells (Ganat et al. 2012; Xia et al. 2016). By contrast, the proteome level changes associated with DA neuron differentiation are not clearly understood. In this report, through proteome analysis, we demonstrated that a number of cytoskeletal proteins were specifically up-regulated during differentiation of hESC-derived neural precursor cells into DA neurons. In addition, we studied their potential roles in the maturation of DA neurons and possible involvement in PD pathogenesis.

## Materials and methods

### Maintenance and differentiation of hESC

Differentiated DA neurons derived from H9 stem cells (established at the University of Wisconsin) were provided by Prof. Yong-Sung Lee (Department of Biochemistry, Hanyang University). Maintenance and *in vitro* differentiation of H9 cells were carried out as described before (Park et al. 2005). Briefly, undifferentiated hESC were propagated on a feeder layer of γ-irradiated CF1-mouse embryonic fibroblasts in ES-medium (DMEM/F-12 media supplemented with 20% knockout serum replacements, non-essential amino acids, mercaptoethanol, antibiotics and bFGF). For maintenance, cells were passaged once a week by dissecting and transferring colonies onto freshly prepared feeder cells. To induce neural precursor cells, undifferentiated cells were detached from the feeder by using collagenase IV and dissociated into small clusters and then re-suspended in serum-free insulin/transferrin/selenium medium containing ascorbic acid (ITSA medium). Feeder cells were replaced with MS5 stromal cells and then MS5 cells over-expressing Shh. Neural precursor cells were grown in the expansion medium (ITSA medium and bFGF) and the resulting spheres were dissociated into single cells by incubating Ca^2+^/Mg^2+^-free HBSS. For differentiation into DA neurons, neural precursor cells were incubated without bFGF and treated with BDNF, GDNF, transforming growth factor-β (TGF-β) and dibutyryl cAMP. Differentiating DA neurons were collected every 3 days and subjected to the proteome analysis.

### Two-dimensional gel electrophoresis (2-DE)

2-DE was carried out using the Multiphor system (Amersham Pharmacia) for IEF and Protean II system (Bio-Rad) for SDS-PAGE. Protein samples (150–200 μg) in 250 μl of solubilization solution (9 M urea, 2% CHAPS, 4 M thiourea, 2% IPG buffer; pH 4–7, 18 mM DDT and bromophenol blue) were loaded onto Immobiline Drystrips (13 cm, pH 4–7) and rehydration was preceded for 12 h at room temperature. IEF was conducted in gradient mode for 1 h at 1000 V, 1 h at 2000 V and 10 h at 8000 V, followed by 8000 V for a total of 65 kVh at 20°C. After the first-dimensional separation, the gel strips were equilibrated for 15 min in equilibration buffer (50 mM Tris-HCl pH 6.8, 6 M urea, 30% glycerol, 2% SDS and bromophenol blue). For the first equilibration, 0.25% DTT was added, and for the second equilibration, 4.5% iodoacetamide was used. SDS-PAGE was carried out in 12% separation gels with constant current of 40 mA/gel. After electrophoresis, the proteins were visualized by silver staining (GE Healthcare) and then the 2-DE images were obtained and analyzed using the Progenesis SameSpots program, v2.0 (Nonlinear Dynamics).

### In-gel trypsin digestion and peptide extraction

Gel pieces containing protein spots were excised from 2-DE gel and incubated in oxidation buffer (15 mM potassium ferricyanide and 50 mM sodium thiosulfate) at room temperature until the spots were destained. After washing, the gels were re-swelled and dehydrated with 100 mM ammonium bicarbonate in 50% acetonitrile. To dehydrate the gels further, acetonitrile was removed and the samples were spun in a Speed-Vac for 5 min at room temperature. After drying, the gel pieces were rehydrated with 20 μl of trypsin solution (20 ng/μl; in 50 mM ammonium bicarbonate) and digestion was performed overnight at 37°C. The tryptic peptides were extracted from the gels and concentrated using a Speed-Vac at room temperature and then mixed with 20 μl of 0.1% formic acid in 3% acetonitrile.

### LC-MS analysis and protein identification

Waters Synapt^TM^ HDMS system coupled with the Waters Nano UPLC system was used for mass spectrometry. Nano LC of tryptic peptides was performed with the Waters Nano UPLC system equipped with a Waters NanoEase Atlantis C_18_ reverse phase column (75 μm × 25 cm). Binary solvent A1 contained 0.1% formic acid in water and binary solvent B1 contained 0.1% formic acid in acetonitrile. Samples (1000 ng per injection) were loaded onto the column and the peptides were eluted with a gradient of 2–40% binary solvent B1 for 120 min at 0.3 μl/min. The lock mass, [Glu^1^]-fibrinopeptide at 400 fmol/ μl, was delivered from the auxiliary pump of the Nano LC system at 0.1 μl /min to the reference sprayer of the NanoLockSpray^TM^ source.

Mass spectrometry analysis of tryptic peptides was performed using Waters Synapt^TM^ HDMS. The mass spectrometer was operated in V-mode for all measurements. All analyses were performed using positive mode Nano ESI using a NanoSpray source. The lock mass channel was sampled every 30 s. The mass spectrometer was calibrated with a [Glu^1^]-fibrinopeptide solution (400 fmol/μl) delivered through the reference sprayer of the NanoLockSpray source. Accurate mass LC-MS data was collected in data-dependent acquisition (DDA) mode. The raw data were processed for database search by using the ProteinLynx Global Server (PLGS) version 2.3 (Waters). The identities of proteins were determined by searching human databases (SWISS-PROT and TREMBL). Ion detection, clustering and normalization were also processed using PLGS.

### RT–PCR analysis

For construction of a cDNA library, total RNAs were isolated from DA neurons using TRI reagent (Molecular Research Center Inc.) and cDNA was synthesized from 5 μg of total RNA using a RT–PCR kit (Thermo Fischer). The primer sets used for RT–PCR analysis were as follows; LMNA (forward: 5′-AGA TGA CCT GCT CCA TCA CC-3′; reverse: 5′-ACA TGA TGC TGC AGT TCT GG-3′), TPM1 (forward: 5′-GAA GTC ACT GGA GGC TCA GG-3′; reverse: 5′-GCT CAG AGA GGT GGG ACA TC-3′), MYL1 (forward: 5′-ACG TGA AGA AAC CTG TGG CT-3′; reverse: 5′-CCT TGT CAA AGA CAC GCA GA-3′), PDI A3 (forward: 5′-CAA CGA GTT TCT CAG GGA GC-3′; reverse: 5′-ATA CGA CTC AAT TCA CCG GC-3′) and actin (forward: 5′-AGA GCT ACG AGC TGC CTG AC-3′; reverse: 5′-CAC CTT CAC CGT TCC AGT TT-3′).

## Results

**Figure 1.**
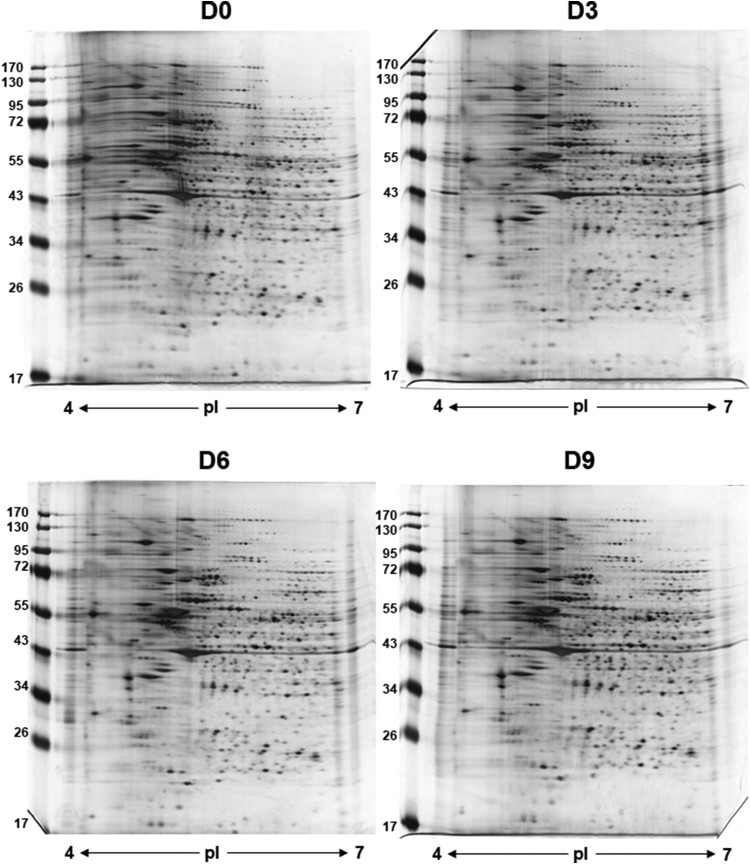
2-DE patterns of soluble proteins of differentiating dopaminergic (DA) neurons derived from hESC. DA neurons were collected every three days from the initiation of differentiation (D0, D3, D6, D9) and 150 - 200 μg of the soluble proteins extracted from the cells were subjected to 2-DE (*pI* 4-7 linear gradient and 12% SDS-PAGE). Images were analyzed and the numbers of resolved protein spots were determined by using the Progenesis SameSpots program (v2.0). While the number of protein spots varied among the samples (778 ∼ 953), the overall protein complexity of DA neurons were consistent. Experiments were conducted in triplicate and the representative 2-DE images are shown here.

**Figure 2.**
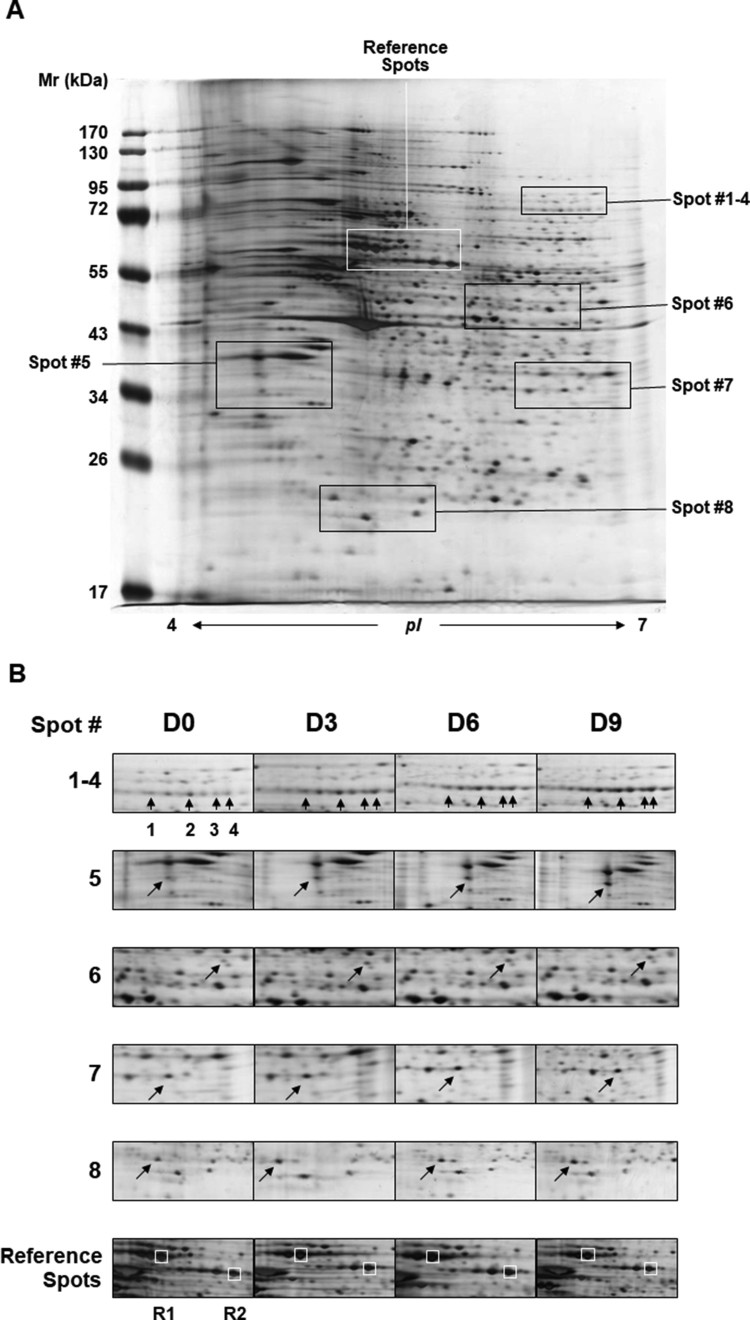
Identification of differentially expressed proteins. (A) Proteins whose expression levels changed by more than two-fold during differentiation were selected from the gels shown in Fig. 1 (the D0 image is used as a reference to depict the location of each spot) and compared for their relative intensities. (B) Eight protein spots were finally selected and subjected to LC/MS spectrometry for identification. Spots #1-4 were revealed as lamin-A/C (LMNA). Spots #5 and #8 were identified as tropomyosin alpha 1 (TPM1) and myosin light chain 1/3 (MYL1), respectively. Spots #6 and #7 are type II cytoskeletal keratin (KRT1). Two landmark spots (R1 and R2), chosen as negative controls, were identified as heat shock protein 60 (HSPD1) and protein disulfide isomerase A3 (PDI A3), respectively (see Table 1 for the details).

**Figure 3.**
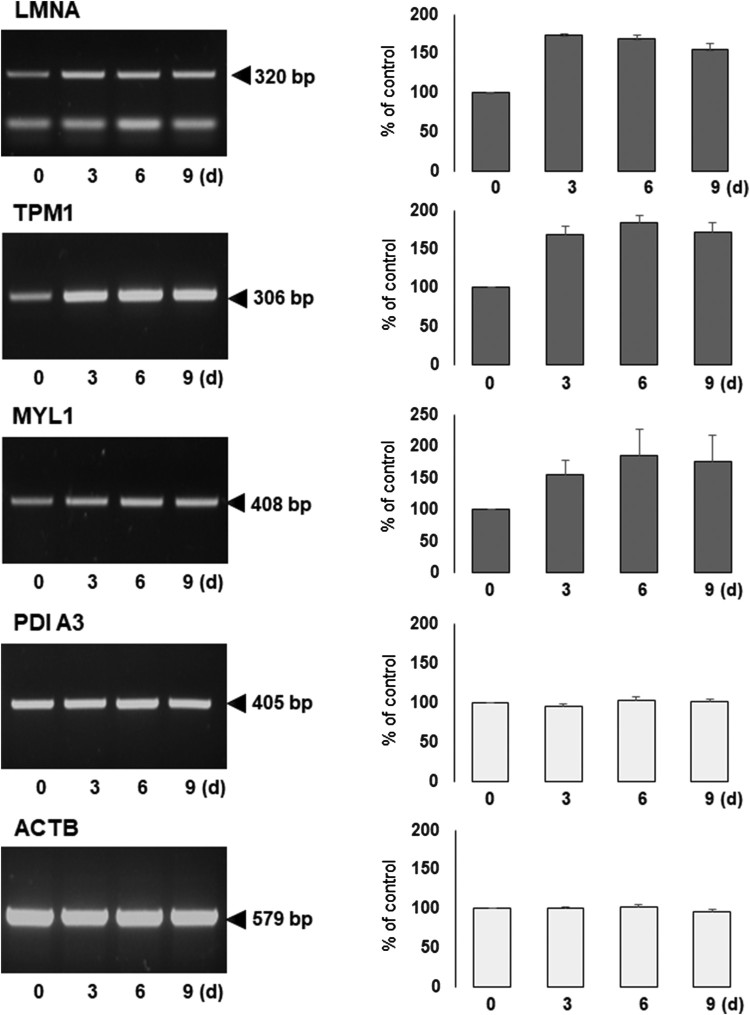
RT–PCR analysis of gene expression of the identified proteins. To verify the results of proteome analysis, four genes (LMNA, TPM1, MYL1 and PDIA3) and β-actin (a loading control) were selected and their relative levels of gene expression (expressed as % of the control, the data from D0) during differentiation were measured by RT-PCR analysis. Experiments were carried out in triplicate and the representative gel images are shown here. The data shown in bar graphs represent mean ± S.E.

To obtain the maximum resolution, we employed the following parameters for proteome profiling throughout the study; pH 4–7 linear gradient, 12% SDS-PAGE and 150–200 μg of soluble proteins. The numbers of resolved protein spots (visualized by silver-staining and determined by image analysis) were as follows; 885 spots (D0), 892 spots (D3), 778 spots (D6) and 953 spots (D9) (Figure 1). Although the numbers of spots varied, no significant change in the complexity of protein patterns was observed and the relative intensities of numerous abundant proteins were mostly consistent among the samples. With the aid of image analysis software, we chose approximately thirty protein spots exhibiting intensity changes by more than 50%. Among them, we selected eight proteins whose expression increased by more than two-fold during differentiation and subjected them to mass spectrometry for the identification (Figure 2). Spots #1–4 were all identified as lamin A/C (LMNA) – these spots are possibly the isoforms or post-translationally modified forms of LMNA since their molecular weights were identical while their isoelectric points were different. Spot #5 and #8 were identified as tropomyosin alpha 1 (TPM1) and myosin light chain 1/3 (MYL1), respectively. Finally, spots #6 and #7 were identified as type II keratin (KRT1). Interestingly all of the identified proteins, except for LMNA, are functionally related to cytoskeletal network. For the reference, we chose two landmark proteins which were identified as heat shock protein 60 (hsp60) and a member of protein disulfide isomerase family (PDI A3) (Table 1). To compare with and validate the proteome results, we measured the gene expression of these proteins. Using RT–PCR analysis, we measured the relative mRNA levels of LMNA, TPM1 and MYL1 together with the landmark protein PDI A3 (β-actin as a loading control). As expected, RT–PCR analysis results were consistent with the proteome data and the gene expression of *LMNA*, *TPM1* and *MYL1* increased nearly two-fold during differentiation whereas the gene expression of *PDI A3* was unchanged (Figure 3).

**Table 1.** Proteins differentially expressed in H9-derived DA neurons during differentiation.

Spot No.	Protein name	Accession No. (UniProtKB)	Coverage (%)	Match	Score	Expression
1	Prelamin-A/C (LMNA)	Q3BDU5	9.65	5	10.869	Up
2	Prelamin-A/C (LMNA)	Q3BDU5	10.26	5	10.869	Up
3	Prelamin-A/C (LMNA)	P02545	11.14	7	11.165	Up
4	Prelamin-A/C (LMNA)	P02545	5.59	3	10.244	Up
5	Tropomyosin alpha 1 chain (TPM1)	P09493	16.90	5	10.472	Up
6	Keratin, type II cytoskeletal 1 (KRT1)	P04264	9.00	6	11.165	Up
7	Keratin, type II cytoskeletal 1 (KRT1)	P04264	11.64	8	11.165	Up
8	Myosin light chain 1/3, skeletal muscle isoform (MYL1)	P06741	24.0	3	10.970	Up
R1	60 kDa heat shock protein, mitochondrial (HSPD1)	P10809	24.25	11	11.165	Landmark
R2	Protein disulfide isomerase A3 (PDIA3)	P30101	7.76	4	11.165	Landmark

**Figure 4.**
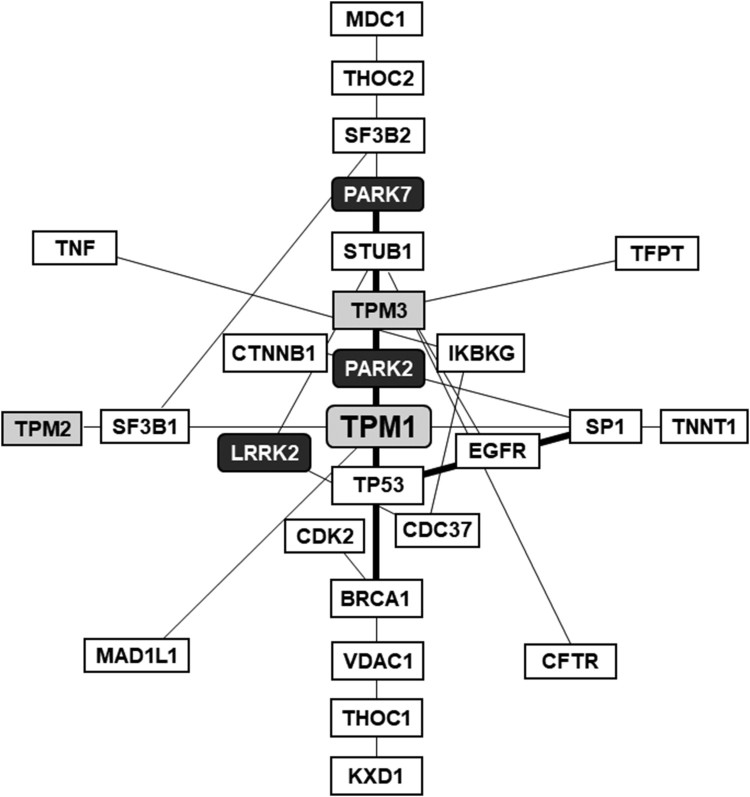
Interaction between TPM1 and PD-related proteins. To determine whether the differentially expressed proteins listed in Table 1 interact with the PD-related proteins, we searched the public database and found that TPM1, MYL1 and LMNA bind to a number of PD-related proteins including parkin, DJ-1 and LRRK2 (see Table 2 for the details). The network diagram shows that TPM1, together with another members of tropomyosin family TPM2 and TPM3 (gray boxes), form protein interaction network with parkin (PARK2), DJ-1 (PARK7) and LRRK2 (PARK8) (black boxes). The diagram was retrieved from BioGRID^3.5^ (minimum evidence = 5).

To investigate a possibility that these proteins are also involved in the pathological mechanism of PD, we searched public databases, such as IntAct Molecular Interaction Database (EMBL-EBI) and Biological General Repository for Interaction Datasets (BioGRID^3.5^), to examine the interaction between these differentially expressed proteins and PD-related proteins (especially *PARK* genes). As summarized in Table 2, TPM1, MYL1 and LMNA are all shown to associate with one or more of PD-related proteins. Of particular interest is TPM1 since this protein interacts with multiple PD-related proteins including parkin (PARK2), DJ-1 (PARK7) and LRRK2 (leucine-rich repeat kinase 2; PARK8) (Figure 4). Likewise, LRRK2 binds to both TPM1 and MYL1 and parkin binds to both TPM1 and LMNA, respectively. Taken together, these findings raise a possibility that these up-regulated proteins not only play roles in the cytoskeletal rearrangement required for neuritogenesis of DA neurons but also involve in PD pathogenesis via their ability to interact with several PD-related proteins.

**Table 2.** Interaction between differentially expressed proteins and PD-related proteins.

Differentially expressed protein	PD-related protein	Database	Reference
Tropomyosin 1 (TPM1)	Parkin (PARK2)	BioGRID	Zanon et al. (2013)
	DJ-1 (PARK7)	IntAct	Malty et al. (2017)
	LRRK2 (PARK8)	BioGRID	Meixner et al. (2011)
Myosin Light Chain 1/3 (MYL1)	LRRK2 (PARK8)	IntAct	Martin et al. (2014)
Prelamin-A/C (LMNA)	Parkin (PARK2)	BioGRID	Zanon et al. (2013)

## Discussion

It is now feasible to generate functional neurons from hESC with a high efficiency (Yasuhara et al. 2017). Continuous efforts are being made to overcome potential tumorigenesis associated with *in vivo* application and to address ethical issues. As animal studies have already demonstrated the therapeutic potentials, clinical trials using hESC-derived DA neurons are currently under development (Man et al. 2018). To ensure the fidelity and efficiency of differentiation, the expression of mid-brain DA neuron specific genes is thus carefully assessed (Kirkeby et al. 2012). A recent study investigating the gene expression on the transcriptome level verified that neuron-specific markers were readily detected in hESC-derived DA neurons differentiated *in vitro*. However, it was also reported that the global gene expression of *in vitro* induced DA neurons differs from that of mid-brain DA neurons *in vivo* (Xia et al. 2016). Another study comparing the gene expression of undifferentiated precursor cells derived from H9 cells and the post-mortem tissue from human substantia nigra (rich in DA neurons and glial cells) revealed that the genes of the mitotic cell cycle are up-regulated in precursor cells whereas the genes involved in the DA neurotransmitter release cycle and central nervous system development are highly expressed in the substantia nigra (Marei et al. 2011). Although those results did not necessarily represent a direct comparison of precursor cells and differentiated DA neurons, they provided insights into the cellular pathways important for dopaminergic differentiation. Since the cell replacement therapy involve transplantation of immature progenitor cells, which then undergo differentiation *in vivo* (Kirkeby et al. 2017), the refinement of differentiation protocol and understanding of molecular changes occurred during neuronal differentiation are therefore crucial for clinical applications.

While the transcriptome analysis of differentiating DA neurons has been extensively conducted, the proteome changes are much less frequently investigated. In fact, there are only a few reports on the proteome changes associated with the differentiation of neuronal cells derived from ESC. A comparative analysis of the proteomes of the undifferentiated hESC and *in vitro* induced mature neurons showed that proteins involved in redox regulation, a number of metabolic enzymes and several proteasome subunits are up-regulated and the chaperones and actin are down-regulated in the mature neurons (Fathi et al. 2014). More relevant to our results, another study analyzing the proteome of the differentiated DA neurons derived from the mesencephalon of embryonic rat reported that actin and vimentin were up-regulated after the differentiation (Weiss et al. 2014). These observations, together with our findings that several proteins involved in cytoskeletal network are up-regulated during differentiation, emphasize the importance of DA neurons’ capability to migrate through the midbrain area. Presumably TPM1 and MYL1, involved in actin cytoskeletal dynamics, play roles in the re-organization of cytoskeletal network required for migration of DA neurons. Indeed, tropomyosins induce neurite outgrowth and regulate neurite branching (Curthoys et al. 2014).

Interactome analysis shows that TPM1, together with another members of tropomyosin family TPM2 and TPM3, forms the interaction network with PD-related proteins including LRRK2, DJ-1 and parkin whose mutations are associated with PD pathogenesis (Figure 4 and Table 2). LRRK2, the most common causative gene of familial PD, is particularly intriguing because this large protein with GTPase and kinase domains is also functionally linked to actin cytoskeleton and known to influence neurite outgrowth. The observation that knock-down of LRRK2 led to the increased expression of tropomyosins (e.g. TPM1, TPM3 and TPM4) hints a pivotal role of LRRK2 and tropomyosin(s) in neuritogenesis (Häbig et al. 2013). Attempts to identify LRRK binding proteins revealed that actin isoforms and several actin regulatory proteins including TPM1, TPM2 and TPM3 were among the interactors (Meixner et al. 2011). Although it was not identified in our proteome study, microarray analysis showed that the expression of *TPM3* was increased by nearly 3-folds during the differentiation of DA neurons (unpublished results). In addition TPM1 was also identified as a genetic modifier of age-at-onset for familial PD (Hill-Burns et al. 2016). Furthermore, MYL1 is reported as a candidate for LRRK2 kinase substrates (Martin et al. 2014). These results together indicate that TPM1 and other actin-associated proteins involved in regulation of actin stability are critical for neuronal migration and neurite extension. In LRRK2-associated familial PD, the perturbation of LRRK2 activities perhaps influences the expression (or stability) of TPM and MYL, which in turn alters actin-based cytoskeletal dynamics and eventually causes the increased degeneration of DA neurons (Meixner et al. 2011). Contrary to LRRK2, the potential influence of interaction between other PD-related proteins (e.g. parkin and DJ-1) and the differentially expressed proteins on PD pathogenesis is less clear. Nevertheless, it can be speculated that the interaction between TPM1 and DJ-1 (PARK7) indicates a functional relationship between mitochondrial dynamics and actin cytoskeletal network (Malty et al. 2017). Likewise, the association of TPM1 and parkin (PARK2), which is also involved in the regulation of mitochondrial dynamics, may suggest a similar role of cytoskeletal proteins with regulatory function in the maintenance of mitochondrial functions.

In conclusion, we report that a number of proteins in cytoskeletal network are specifically up-regulated during the differentiation of DA neurons derived from H9 cell line. These findings confirm the importance of actin-based cytoskeletal dynamics for neuronal migration and neuritogenesis, which are crucial for neural development. In addition, the findings that these up-regulated proteins, especially TPM1, bind to several PD-related proteins raise a possibility that the identified proteins also play roles in the PD pathogenesis through their roles in the regulation of actin cytoskeletal dynamics.
